# Draft Genome Sequence of *Actinomyces odontolyticus* subsp. *actinosynbacter* Strain XH001, the Basibiont of an Oral TM7 Epibiont

**DOI:** 10.1128/genomeA.01685-15

**Published:** 2016-02-04

**Authors:** Jeffrey S. McLean, Quanhui Liu, Batbileg Bor, Joseph K. Bedree, Lujia Cen, Michael Watling, Thao T. To, Roger E. Bumgarner, Xuesong He, Wenyuan Shi

**Affiliations:** a Department of Periodontics, University of Washington, Seattle, Washington, USA; b School of Dentistry, University of California Los Angeles, Los Angeles, California, USA; c Department of Microbiology, University of Washington, Seattle, Washington, USA

## Abstract

Here, we present the draft genome sequence of *Actinomyces odontolyticus* subsp. *actinosynbacter* strain XH001, isolated from the human oral cavity. Uniquely, it was discovered as a host bacterium to the ultrasmall epibiont TM7x, which is the first cultivated member of “*Candidatus* Saccharibacteria” (formerly candidate phylum TM7).

## GENOME ANNOUNCEMENT

Although *Actinomyces odontolyticus* is a commensal oral species, it is an opportunistic pathogen that has been linked to many diseases, most notably its association with actinomycosis, the formation of painful abscesses in the mouth, lungs, or gastrointestinal tract (1). Furthermore, oral *Actinomyces* spp. have been linked to childhood caries, periodontitis, and human oral carcinomas (2–4). A newly reported interaction between an obligate ultrasmall epibiont, TM7x (a recently cultivated member of “*Candidatus* Saccharibacteria,” formerly candidate phylum TM7), and its basibiont *A. odontolyticus* subsp. *actinosynbacter* strain XH001 (5) provides a great system to study parasitic epibiont symbiosis in the bacterial kingdom. The genome presented here from the isolated strain derived from human saliva (6, 7) will enable further research into this unique interaction.

*A. odontolyticus* subsp. *actinosynbacter* strain XH001 was cultured in brain heart infusion (BHI) medium and incubated at 37°C under microaerobic conditions until exponential phase was reached. Genomic DNA was extracted using the Epicentre MasterPure DNA purification kit. The complete genome sequence was determined via Illumina sequencing using paired-end 300-bp reads. All quality-trimmed reads were *de novo* assembled using SPAdes version 3.61 (8, 9).

The draft genome is 2,336,127 bp and assembled into 5 contigs, with an overall G+C content of 65.9%. Gene annotation using the Prokaryotic Genome Automatic Annotation Pipeline (PGAAP) provided by National Center for Biotechnology Information (NCBI) identified a total of 1,998 genes, consisting of 1,936 coding sequences, 49 tRNAs, and 6 rRNAs. A set of 49 single-copy genes was extracted and aligned to 100 sequenced genomes using the Department of Energy Systems Biology Knowledgebase (KBase; http://kbase.us). The resulting likelihood-based tree was built from the 49 concatenated genes and indicated relatedness to the sequenced species *A. odontolyticus* and *Actinomyces* sp. ICM39; however, the closest sequenced genome, *A. odontolyticus* F0309, produced an average nucleotide identity of only 93.7% (10). Using an additional method described for the delineation of species using 40 universal marker genes (11), XH001 was just above the cutoff for the *A. odontolyticus* species. These results support that this strain conservatively represents a subspecies of *A. odontolyticus*, and the name “*A. odontolyticus* subsp. *actinosynbacter* strain XH001” is proposed. The report by Bor et al. (5) presents further physiological characteristics of this strain.

### Nucleotide sequence accession numbers.

This whole-genome shotgun project has been deposited at DDBJ/EMBL/GenBank under the accession no. LLVT00000000. The version described in this paper is version LLVT00000000.1.
